# A Multidisciplinary Aesthetic Treatment Approach for Peg Lateral of the Maxillary Incisors

**DOI:** 10.7759/cureus.29184

**Published:** 2022-09-15

**Authors:** Shruti Rathi, Purva Dhannawat, Rizwan Gilani, Rozina Vishnani

**Affiliations:** 1 Orthodontics and Dentofacial Orthopaedics, Sharad Pawar Dental College, Datta Meghe Institute of Medical Sciences (Deemed University), Wardha, IND; 2 Oral and Maxillofacial Surgery, Sharad Pawar Dental College, Datta Meghe Institute of Medical Sciences (Deemed University), Wardha, IND

**Keywords:** peg lateral, malocclusion, aesthetics, small teeth, hypodontia

## Abstract

Developmental anomaly of the maxillary lateral incisors most commonly leads to the occurrence of peg lateral. It is a variant of microdontia where the lateral incisors are smaller than the normal size. This appears as unilaterally or bilaterally. This condition is characterised by the converging of the mesial and distal surfaces forming a cone shape. A variety of treatment options exist for this anomaly including orthodontic treatment, restorative technique and veneer. This case report deals with an individual presenting with peg lateral of the maxillary arch along with midline diastema. The multidisciplinary treatment protocol of orthodontic treatment involving minor tooth movement and space closure in conjunction with a restorative technique for correction was preferred.

## Introduction

The teeth that are smaller than usual are referred to as microdontia. The lateral incisors of the upper arch are one of the most typical sites for localised microdontia sometimes known as peg laterals. An undersized maxillary lateral incisor is a tooth development defect that is identified by a change in crown morphology. The teeth typically have a narrower incisal dimension and a smaller mesial-distal diameter, with a sharp convergence of the proximal surfaces. The word is typically used to refer to second incisors in which the middle lobe calcifies during development.

The teeth size is mostly determined by genetics, varies by race and may also be brought on by endocrine disorders. The prevalence ranges from 0.8% to 8.4% of the population in the majority of studies [1]. It is not reasonable to state that endocrine problems alter the shape or size of the tooth's crown unless these effects happen during morpho-differentiation, in uterine life or during the first year of life. However, later period disruptions may change the root's size and shape. A peg-shaped or otherwise deformed tooth with the first two layers of tooth that may be normal in structure might result from morpho-differentiation disturbances that impact the tooth's shape and size without compromising the tooth's function or the function of ameloblasts and odontoblasts. Function, aesthetics, the requirement for extractions, the position of the canines and the possibility of coordinating restorative and orthodontic therapy should all be considered when deciding on treatment care plans [2].

Treatment options

The treatment options include i) extraction and repositioning of the canines through orthodontic movement followed by recontouring to simulate lateral incisors, ii) extraction and replacement with single tooth implant restorations or fixed partial dentures (FPDs), iii) retention of the restoration peg lateral incisors to develop normal tooth morphology and iv) restorative techniques.

Building direct composite layers

Acid etch-retained composite is becoming more popular as a reversible tooth addition. This approach allows for the quick and straightforward modification of the morphology of small teeth [3]. However, previous chemically cured composite materials had the disadvantages of low abrasion resistance, staining and short working durations. The wear resistance and command set of the newest light-cured hybrid and microfilled composite materials have grown, allowing for gradual buildups.

Composite indirect veneers

Heymann described the use of indirect laboratory-produced composite veneers in the treatment of unattractive anterior teeth in young adolescent patients [4]. These restorations are made with a microfilled laboratory composite that is treated to a secondary or super curing cycle that includes light, heat and pressure. Because of the more intense curing conditions, the materials have better physical qualities than their direct light-cured counterpart [5]. It has been observed that surface glaze had been lost during a two-year period, and the restorations were subject to chipping and brittle fracture when exposed to significant functional or biting force.

Acrylic laminate veneers

Acrylic laminate veneers, whether prefabricated or custom-made, can be bonded to etched enamel using composite resin cement. However, this technique suffers from the disadvantages of poor bonding between the acrylic and composite cement and poor abrasion resistance of the acrylic. This case report details the care of a patient who had bilateral peg-shaped incisors.

## Case presentation

**Figure 1:**
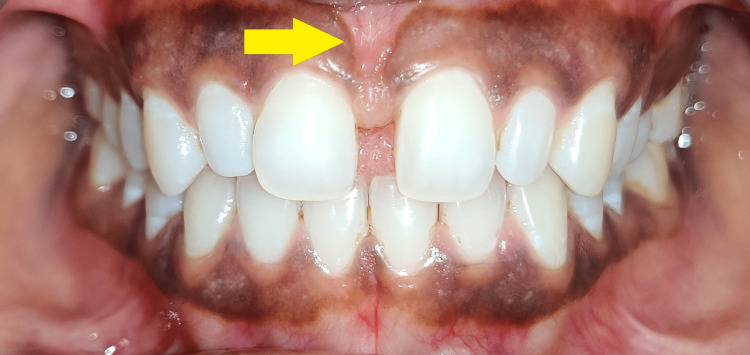
Intraoral photograph (frontal view) Midline diastema along with high frenum attachment

A 22-year-old female presented to the department of orthodontics with a complaint of a small gap in her upper central incisors. Her face was symmetrical, with competent lips and an ordinary grin line. The patient's medical history revealed no systemic illnesses. An evaluation revealed a midline diastema and a high frenum attachment (Figure 1).

**Figure 2:**
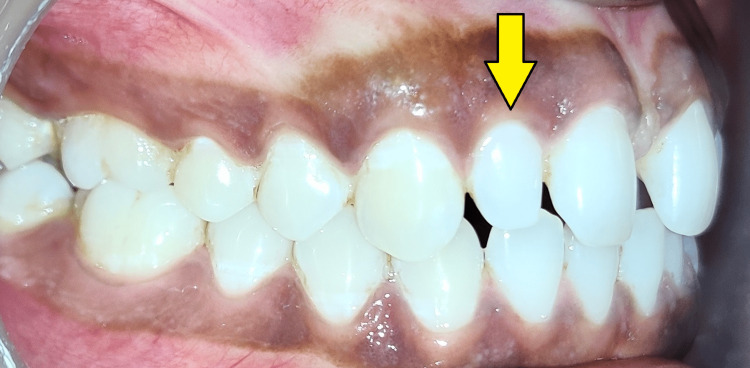
Intraoral photograph (right lateral view) Peg-shaped lateral incisor is evident

**Figure 3:**
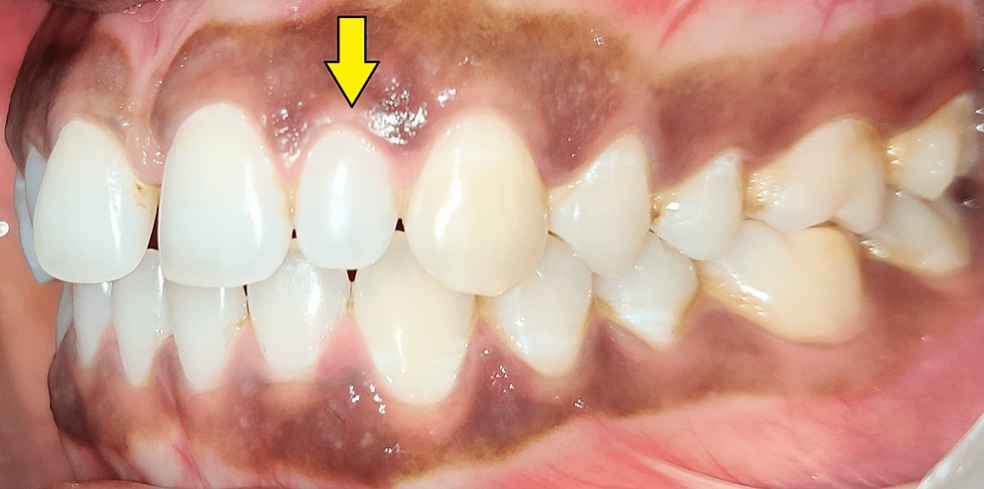
Intraoral photograph (left lateral view) Peg-shaped lateral incisor is evident

It was also observed that the presence of peg-shaped lateral incisors bilaterally was evident. On both sides, the patient had a slightly elevated overjet and a normal overbite, as well as molar and canine to be in class I relationship as seen in Figure 2 and Figure 3.

The treatment goal was to close the midline diastema and correct the peg lateral in order to improve facial aesthetics and create a symmetrical smile.

Orthodontic phase

The preferred plan of treatment was frenectomy followed by space closure by continuous arch mechanics in both the upper and lower arch. To the maxillary and mandibular arches, readjusted edgewise appliances with a 0.022-0.028 slot (Mclaughlin-Bennett-Trevisi {MBT} prescription) were bonded. Nickle titanium (NiTi) wire measuring 0.016 inch functioned as the initial levelling and alignment wire, followed by rectangular 0.016×0.022 NiTi and 0.019×0.025 NiTi. Both arches had stainless steel wire measuring 0.019-0.025 inches posted to prepare them for retraction. Continuous arch mechanics were used to perform en masse retraction in the upper and lower arches.

**Figure 4:**
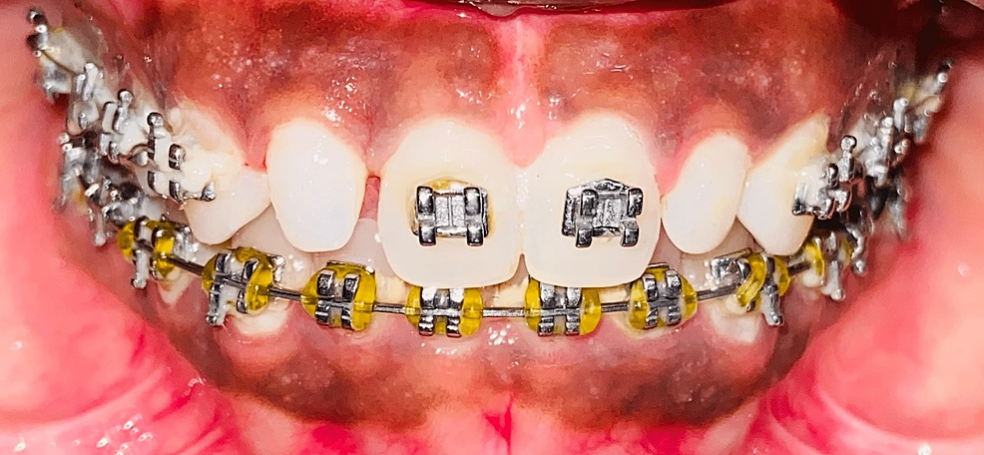
Midline closure After frenectomy, the goal was to close the midline diastema

Under local anaesthesia, frenectomy was performed with a number 11 Bard Parker blade. In this method, lateral incisions were made to the depth of the underlying bone on either side of the frenum. On either side of the excised tissue, sutures were used to mark the free tissue boundaries before periodontal pack was applied for a week. The patient was instructed to come back for suture removal and routine follow-up after a week. During the course of the patient's four-month follow-up, the midline diastema spontaneously closed, leading to a noticeable aesthetic improvement as seen in Figure 4.

The artistic phase

This phase of treatment was performed in the department of orthodontics. The first step in any restoration is to determine shade and opacity with the help of mock-ups of composite resin. This is a technique where different materials are placed on the desired tooth and spot cured. Composite has good handling properties and an excellent lustre but is very translucent. A natural tooth exhibits variation of shade along with depth, especially in young people where the first layer of tooth is translucent. Artists achieve subtlety and a sense of three dimensions by painting in increments, with undercoats effectively casting shadows through succeeding layers. In this case, a similar methodology was used with the tooth built up in stages [6].

**Figure 5:**
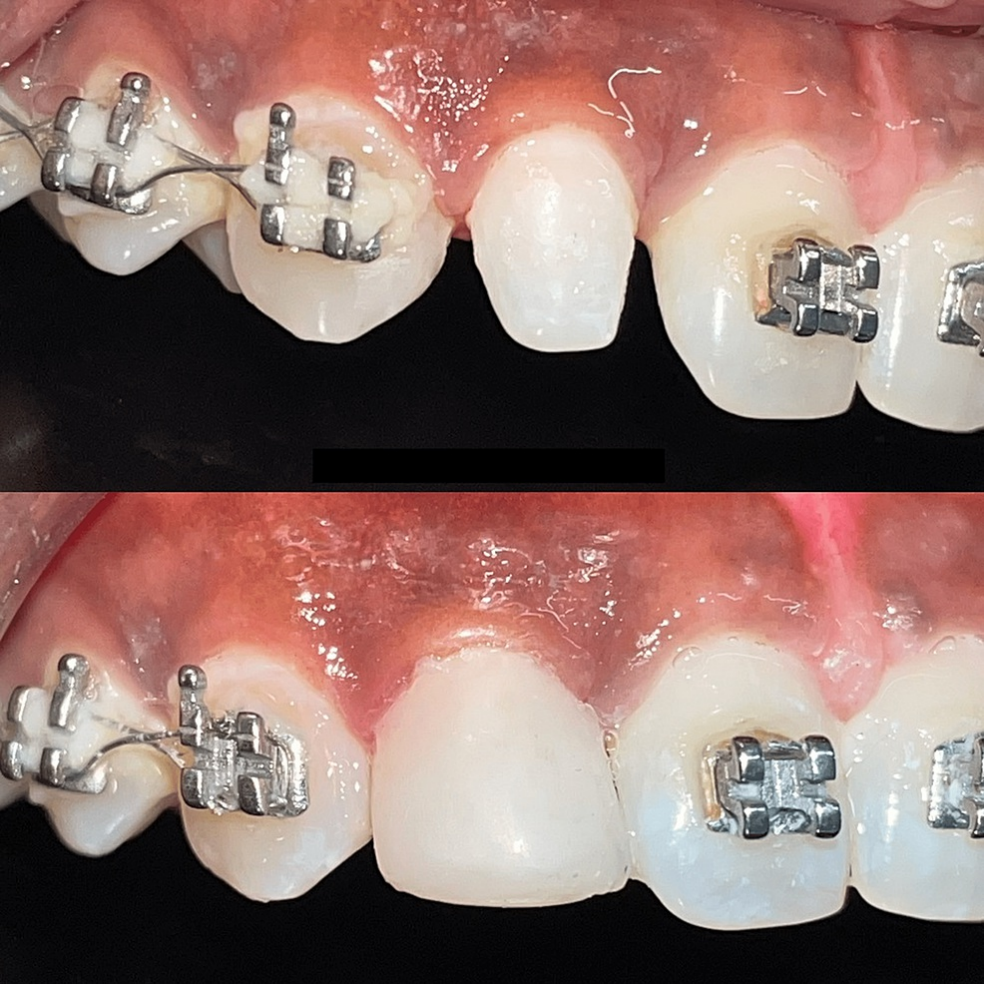
Intraoral photograph (right lateral view) A comparative view of pre- and post-buildup of the lateral incisor

**Figure 6:**
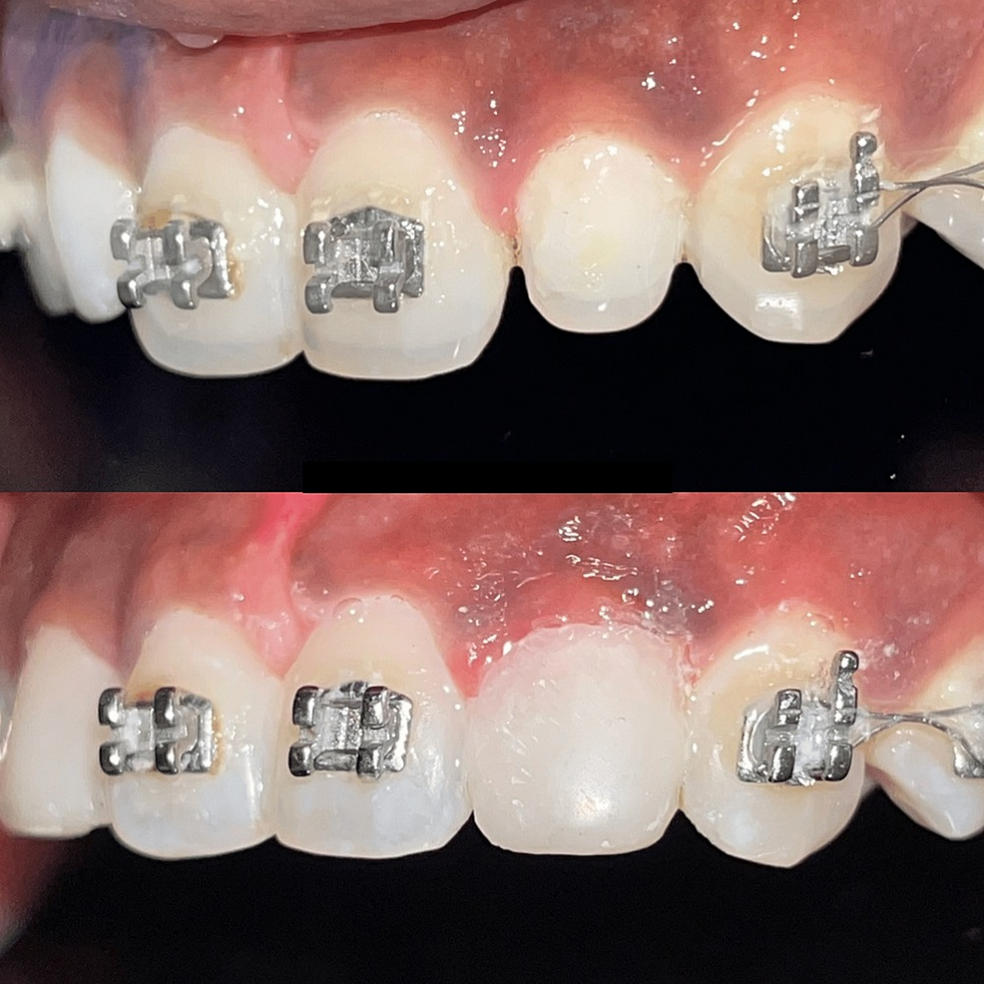
Intraoral photograph (left lateral view) A comparative view of the pre- and post-buildup of the lateral incisor

A thin layer of composite was applied and cured on the labial in the cervical third. Then, next increment was added to the centre before curing, and minute indentations were produced using a probe tip. This first layer was spread with resin glue, blasted with air and left uncured to aid the flow of the subsequent layer and prevent voids. Discrepancies were fixed with flowable composite, which was then dried under a glycerine wash to prevent any air-inhibited layer. After that, discs, friction grip (FG) diamond burs and abrasive strips were used to shape the buildup. To produce undulations and scatter light, tungsten carbide bursts in the shape of footballs were used. The surface was then polished using rubber cones and long, straight multi-fluted burs as seen in Figure 5 and Figure 6. The importance of good plaque control around the margins was emphasised, together with the need for regular review and maintenance.

**Figure 7:**
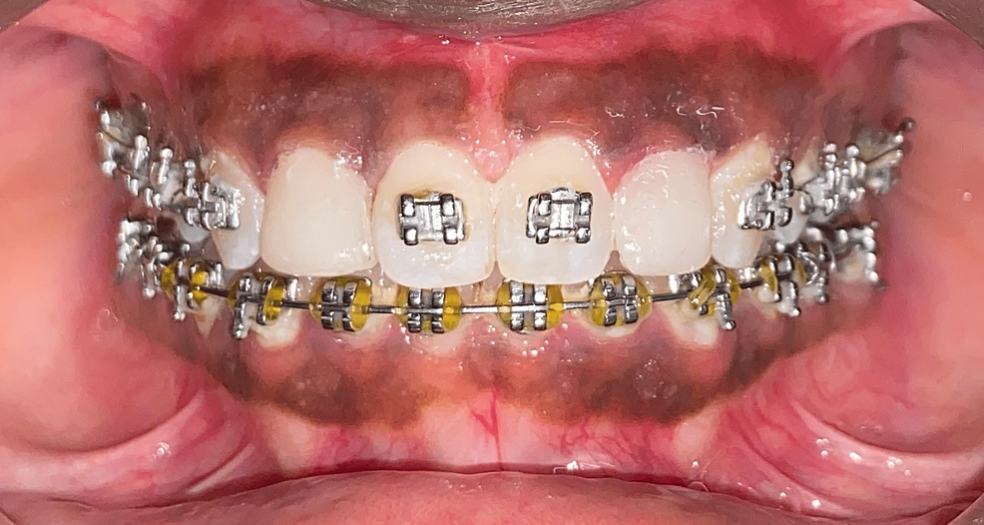
Intraoral photograph (frontal view) The closure of midline diastema along with the lateral incisors having buildup gives an aesthetic smile to the patient

Figure 7 depicts the intraoral frontal view where the incisors are aesthetically pleasant. After this, the orthodontic treatment was completed, and the patient was given a retainer.

## Discussion

Between 2% and 5% of the general population have lateral incisors that are peg-shaped, and females are slightly more likely than males to have them. However, several investigations have found that their bilateral incidence is slightly more common than their unilateral occurrence. They are often distributed evenly on the right and left, unilaterally or bilaterally. When peg-shaped laterals erupt in the mouth, the patient may be upset because their teeth are not ideal or are too little in comparison to the other anterior teeth [7]. The diagnosis of a peg lateral is usually made clinically. To resolve the condition, orthodontic treatment, direct composite bonding onto peg laterals, indirect composite placement, porcelain bonded to metal crowns, crowns bonded to teeth directly, lengthening of crown to improve gingival heights before direct bonding, extractions and implant may be used. The current case study illustrates how orthodontics along with restorative dentistry can interact to produce harmonies in the form of optimal symmetry, proportion and aesthetics [8]. Because hypodontia can create aesthetic, physiological and functional issues, early identification and clinical care of the disorder are crucial [9]. Orthodontic treatment may eliminate some of the periodontal and restorative issues that could occur in these patients as adults, preserving a range of treatment options in the future [10].

## Conclusions

A multidisciplinary approach in treatment planning and performance, as well as the use of restorative techniques, enhances a conservative yet very aesthetic finishing result. A combination treatment approach was preferred to correct a defect that resulted from diastema and peg-shaped lateral incisors. Minor tooth movement and composite buildup on the lateral incisors were completed. In this clinical situation, the restorative treatment benefited from the orthodontic correction of local tooth malposition.
